# High Prevalence of Vitamin D Deficiency in Infertile Women Referring for Assisted Reproduction

**DOI:** 10.3390/nu7125516

**Published:** 2015-12-02

**Authors:** Luca Pagliardini, Paola Vigano’, Michela Molgora, Paola Persico, Andrea Salonia, Simona Helda Vailati, Alessio Paffoni, Edgardo Somigliana, Enrico Papaleo, Massimo Candiani

**Affiliations:** 1 Division of Genetics and Cell Biology, IRCCS San Raffaele Scientific Institute, Milano 20132, Italy; pagliardini.luca@hsr.it; 2 Obstetrics and Gynecology Unit, IRCCS San Raffaele Scientific Institute, Milano 20132, Italy; molgora.michela@hsr.it (M.M.); persico.paola@hsr.it (P.P.); vailati.simonahelda@hsr.it (S.H.V.); papaleo.enrico@hsr.it (E.P.); candiani.massimo@hsr.it (M.C.); 3 Division of Experimental Oncology, URI-Urological Research Institute, University Vita-Salute San Raffaele, Milano 20132, Italy; salonia.andrea@hsr.it; 4 Department of Obstetrics, Gynecology and Neonatology, Fondazione Cà Granda, Ospedale Maggiore Policlinico, Milano 20122, Italy; alessio.paffoni@policlinico.mi.it (A.P.); dadosomigliana@yahoo.it (E.S.)

**Keywords:** vitamin D, assisted reproduction, infertility, 25(OH)D, endometriosis

## Abstract

A comprehensive analysis of the vitamin D status of infertile women is the first step in understanding hypovitaminosis impact on reproductive potential. We sought to determine vitamin D profiles of women attending an infertility center and to investigate non-dietary determinants of vitamin D status in this population. In this cross-sectional analysis, a cohort of 1072 women (mean age ± standard deviation 36.3 ± 4.4 years) attending an academic infertility center was used to examine serum 25-hydroxy-vitamin D (25(OH)D) levels in relation to demographic characteristics, seasons and general health risk factors. Both unadjusted and adjusted levels of serum 25(OH)D were examined. Median 25(OH)D concentration was below 30 ng/mL for 89% of the entire year. Over the whole year, 6.5% of patients had 25(OH)D levels ≤10 ng/mL, 40.1% ≤20 ng/mL, and 77.4% ≤30 ng/mL. Global solar radiation was weakly correlated with 25(OH)D levels. At multivariable analysis, 25(OH)D levels were inversely associated with BMI; conversely, 25(OH)D levels were positively associated with height and endometriosis history. Serum 25(OH)D levels are highly deficient in women seeking medical help for couple’s infertility. Levels are significantly associated with body composition, seasonal modifications and causes of infertility. Importantly, this deficiency status may last during pregnancy with more severe consequences.

## 1. Introduction

Animal and human studies suggest that vitamin D is involved in many processes of the human reproductive system in both genders. Of those, some evidence come from the Assisted Reproduction Technology (ART) [1,2]. ART represents a valuable model to draw inferences on vitamin D deficiency in specific aspects of human fertility as it allows the separate evaluation of the various steps of the reproductive process, from sperm function to folliculogenesis to embryo implantation. 

Six original articles have investigated the association between serum levels of 25-hydroxy-vitamin D (25(OH)D), the storage form of the vitamin, and pregnancy rates in ART cycles with controversial results. For instance, Rudick *et al*. [3] observed that serum 25(OH)D levels were significantly related to implantation, clinical pregnancy, and live birth rates, although opposite trends were found according to patients’ ethnicity being critical in non-Hispanic whites, but not in the Asian ethnicity. In a second study [4], the same authors examined serum 25(OH)D concentration among recipients of oocyte donation, finding a positive association between vitamin D status and clinical pregnancy rate and suggesting the specific effect of 25(OH)D levels on ART outcomes to be mediated by endometrial receptivity rather than by ovarian stimulation or embryo parameters. To this regard, it should be emphasized that both cyclic and early pregnant endometrium represents an extra-renal site of vitamin D synthesis; moreover, the effect of vitamin D at the uterine level is thought to be exerted via the vitamin D receptor (VDR) through either the regulation of target genes or the hormonal effects on the local immune responses [5]. In patients who underwent single embryo transfer at blastocyst stage, vitamin D deficiency (<20 ng/mL) emerged as an independent predictor of lower clinical pregnancy rates as compared with non-deficient women [6]. In contrast, in the largest sample analyzed so far, Franasiak *et al*. [7] showed that vitamin D status was unrelated to pregnancy outcomes in women undergoing euploid blastocyst transfer. Two Iranian studies did not confirm any influence of serum 25(OH)D levels in terms of pregnancy rate [8,9], but these latter analyses were exposed to a significant risk of type II error given the extremely low proportion of women with sufficient 25(OH)D levels. Using a different approach, Farzadi *et al.* reported a correlation between follicular fluid 25(OH)D concentration and assisted reproductive outcomes in an Iranian population [10].

Results from a further cross-sectional prospective study supported the role of vitamin D in terms of female fertility [1]; indeed the odds ratio (OR) for clinical pregnancy in women with vitamin D greater than 20 ng/mL was 2.15 (95% CI 1.23–3.77). Likewise, the group with serum levels >30 ng/mL (sufficient vitamin D) had the highest chances of pregnancy. A similar figure was observed when considering the implantation rate (OR 1.91, 95% CI 1.20–3.05). Finally, women with sufficient 25(OH)D concentration had a significantly higher chance of obtaining top quality embryos and to transfer at the blastocyst stage, thus generally supporting a favorable effect of vitamin D at both ovarian and endometrial level [1].

Based on these observations, we comprehensively analyzed vitamin D status of couples attending a single academic infertile center. Such an evaluation would provide an estimation of a cross-sectional epidemiological magnitude of vitamin-D deficiency, while forming the basis for interventions to address the deleterious consequences of vitamin D deficiency-related infertility problems. We aimed to (i) determine the baseline vitamin D profiles of women attending an infertility center; and, (ii) investigate the non-dietary determinants of vitamin D status in the same cohort. We hypothesized that (i) a high proportion of our cohort would be vitamin D deficient; and (ii) vitamin D status in infertile women mainly relies on social habits, degree of sun exposure and health risk factors. This issue is also of particular clinical interest considering the growing and consistent evidence supporting the idea that vitamin D deficiency may influence birth outcomes and may be associated with relevant obstetrics complications [11,12,13,14].

## 2. Experimental Section

### 2.1. Participants

The analyses of this cross-sectional study were based on the medical records of a cohort of 1253 Caucasian-European female patients assessed at a single academic centre for couple’s infertility (non-interracial infertile couples only) between January 2011 and December 2013. According to the World Health Organisation (WHO) criteria, infertility is defined as not conceiving a pregnancy after at least 12 months of unprotected intercourse regardless of whether or not a pregnancy ultimately occurred [15]. Primary infertility is defined when a couple has never been able to conceive [16]. Secondary infertility is defined as the inability to conceive following a previous pregnancy [16]. Male factor infertility was defined after a comprehensive diagnostic evaluation of all the female partners. All women had a normal basic fertility work up, including hysterosonography, hysterosalpingography or laparoscopy. Patients were enrolled if they had serum 25(OH)D level obtained. The serum 25(OH)D concentration estimations were conducted as part of a routine biochemical laboratory testing in the couples. Overall, patients were excluded if they were receiving pharmacological vitamin D or calcium supplementation prior to the date of 25(OH)D concentration measurement. Patients were also excluded if they were receiving medications potentially affecting vitamin D metabolism or had a concurrent diagnosis of a disorder that may impact on calcium or vitamin D metabolism, such as bone, parathyroid gland, kidney and liver disorders. Diagnosis of endometriosis was obtained by laparoscopy through the evidence of peritoneal implants, ovarian cysts or infiltrating nodules or by ultrasound through the evidence of ovarian cysts or deep peritoneal nodules [17]. Poor ovarian response has been defined according to the Bologna criteria of the European Society of Human Reproduction and Embryology (ESHRE) consensus [18]. Polycystic Ovarian Syndrome (PCOS) has been defined according to the 2003 Rotterdam criteria [19]. Male factor infertility was defined after a comprehensive diagnostic evaluation, thus including at least two consecutive pathologic semen analyses according to the WHO guidelines [20].

All women undergoing in vitro fertilization (IVF) or intracytoplasmic sperm injection (ICSI) cycles routinely provide informed consent for their clinical data and anonymised records to be used for general research purposes. Data collection followed the principles outlined in the Declaration of Helsinki; all patients had signed an informed consent agreeing to deliver their own anonymous information for future studies. Approval from San Raffaele’s Hospital Ethics Committee was obtained.

### 2.2. Variables Measured

Height and weight were measured with standardized protocols. A wall mounted stadiometer measured height to the nearest 0.5 cm and weight was measured using calibrated scales to the nearest 0.1 kg. Body mass index (BMI) was then calculated. The quantitative detection of 25(OH)D levels was performed using a commercially available kit based on a chemiluminescence technology (DiaSorin, Inc. Corp., Stillwater, MN, USA). The intra-assay and inter-assay coefficient of variations were 10% and 15%, respectively. Concentrations of 25(OH)D were described according to the Endocrine Society Clinical Practice Guideline [21]: deficiency was defined as a 25(OH)D concentration below 20 ng/mL and vitamin D insufficiency as a 25(OH)D concentration of 21–29 ng/mL. However, it is necessary to mention that these definitions are not universally accepted. In fact, after considering data for bone health, the Institute of Medicine (IOM) Committee’s 2011 Report on Dietary Reference Intakes for Calcium and Vitamin D [22] defined that a serum 25(OH)D level of 20 ng/mL is adequate for more than 97.5% of the general population from the United States and Canada, while 16 ng/mL 25(OH)D level is adequate for 50% of the general population. The Endocrine Society definitions were used in the text only for descriptive purpose while for the statistical analysis a threshold-independent method was used. Determinations of follicular phase serum estradiol (E2), anti-müllerian hormone (AMH), follicle-stimulating hormone (FSH), luteinizing hormone (LH), prolactin (PRL) and thyroid-stimulating hormone (TSH) levels were obtained within 90 days from the 25(OH)D concentration dosage. Data for number of cigarettes currently smoked per day was self-reported. Latitude was calculated based on patients’ residence. Information about daily global solar radiation from nine different locations in the Lombardy region for the whole study period was obtained from the Regional Environmental Protection Agency (ARPA) [23] and the mean value of the nine different locations was calculated.

### 2.3. Curves

Seasonal levels of 25(OH)D and global solar radiation were represented for days of the year without taking into account year of dosage since no significant variation was observed for these variables among the three years of the study. Values for 25(OH)D levels and global solar radiation were ordered based on the day of the year of dosage/measurement and plotted as the moving median of a 30-day large window shifting by five-days intervals. The same sample windows were used for representing prevalence of patients below the threshold values of 30 ng/mL, 20 ng/mL and 10 ng/mL.

2.4 Statistical Analyses

Data are presented as means ± standard deviation (SD) or medians (ranges). For the specific purpose of overcoming the need for a threshold to define vitamin D deficiency and at the same time make it possible to compare 25(OH)D concentration measures performed at different times of the year, we decided to use the following population-based method: (i) 25(OH)D concentration measures were ordered based on the day of the year of dosage; (ii) *Z*-scores values, defined as the distance in units of standard deviations of each 25(OH)D concentration value from the population’s mean of a 30 days-large window centered on the date of dosage, were calculated. Due to their non-perfect Gaussian distribution, *Z*-scores were subsequently transformed into a percentile rank and these values were used in a univariable linear regression analysis to determine the possible factors influencing vitamin D status. Coefficients resulting from the regression analysis (B) are expressed as the difference in normalized vitamin D concentration (*Z*-scores) percentiles due to a change of one unit in the independent variable; coefficients for categorical variable are compared to the reference category indicated by B equal to 0. Variables showing a significant association with 25(OH)D concentration at the univariable analysis and variables known to be potentially associated with 25(OH)D status (age, weight, high, latitude, presence of leiomyomas) were included in a multivariable linear regression model. All categorical variables were included in the model as *n −* 1 dummy variables; for causes of infertility, the “mixed” variable was excluded from the analysis due to the overlap with the other variables. Data for prevalence of patients with 25(OH)D concentration less than 10, 20 and 30 ng/mL for different trimesters was compared using the Fisher’s exact test and the first trimester was used as reference group for odds ratio (OR) calculation. Statistical tests were performed using SPSS v.19 (IBM Corp., Armonk, NY, USA). All tests were two sided, with a significance level set at 0.05.

## 3. Results

### 3.1. Seasonal Vitamin D Status in Women Attending the Infertility Center

Complete data collection was available for 1072 women. Table 1 lists the characteristics and the descriptive statistics of the entire cohort of patients. Patients were born in Italy and 4.6% were obese. More common causes of infertility were represented by male infertility and infertility of unknown cause. Almost one fourth of patients had more than one cause of infertility.

**Table 1:** Descriptive characteristics of women included in the study. Latitude was converted in decimal form. FSH, follicle-stimulating hormone; LH, luteinizing hormone; E2, estradiol; PRL, prolactin; TSH, thyroid-stimulating hormone; AMH, anti-müllerian hormone; SD, standard deviation.

Parameter	Mean (SD)/Number (%)	Range
25-hydroxyvitamin D (ng/mL)	24.4 (13.0)	2–101
Age (Years)	36.3 (4.4)	20–47
Latitude (degree)	45.2 (1.3)	37–47
Weight (Kg)	60.4 (10.3)	34–110
Height (cm)	164.4 (6.2)	143–185
Body Mass Index (Kg/m^2^)	22.4 (3.6)	14–40
Current smoking (No. cigarettes/day)		
0	836 (78.0%)	
1–15	173 (16.1%)	
>15	63 (5.9%)	
FSH (IU/L)	8.4 (6.5)	0–41
LH (IU/L)	5.9 (4.8)	0–21
E2 (pg/mL)	49.7 (23.2)	9–129
PRL (ng/mL)	14.1 (7.6)	2–66
TSH (µU/mL)	2.2 (1.2)	0–7
AMH (ng/mL)	2.2 (2.4)	0–15
Antral follicles count (*n*)	10.1 (6.1)	0–40
Type of infertility		
Primary	745 (69.5%)	
Secondary	327 (30.5%)	
Cause of Infertility		
Male factor	175 (16.3%)	
Tubal factor	66 (6.2%)	
Poor ovarian response	140 (13.1%)	
Polycystic Ovarian Syndrome	161 (15.0%)	
Endometriosis	75 (7.0%)	
Idiopathic	196 (18.2%)	
Mixed	259 (24.2%)	

**Figure 1:**
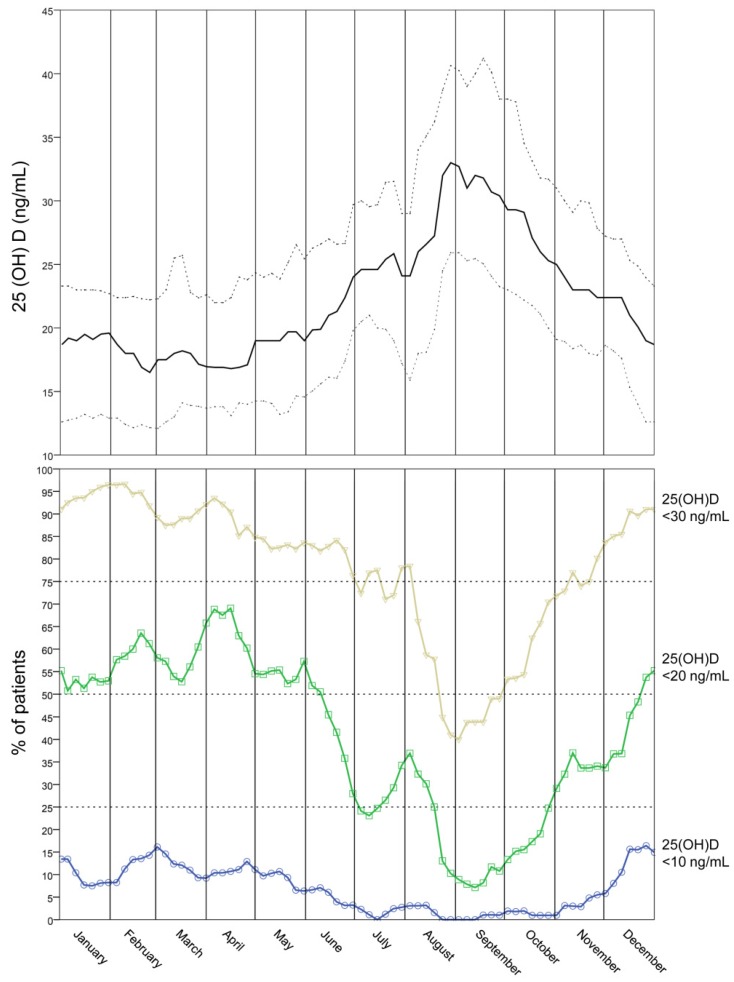
Annual serum 25(OH)D levels. Upper panel shows the moving median for annual serum 25(OH)D concentration for the entire cohort of women. Lower and upper dotted lines represent 25th and 75th percentiles, respectively. Lower panel graphically depicts the relative proportion of patients with serum 25(OH)D concentrations below the threshold values of 30 ng/mL (yellow open triangles), 20 ng/mL (green open squares) and 10 ng/mL (blue open circles) as a function of the monthly period.

Figure 1 (upper panel) depicts the curve for annual serum 25(OH)D levels (median, 25th and 75th percentiles) for the entire cohort of women. Modifications in serum levels of 25(OH)D followed a seasonal cycle. The lowest median serum 25(OH) concentration occurred at the end of February (16.5 ng/mL) and the highest (33.0 ng/mL) at the end of August. Overall, the median value of serum 25(OH)D concentrations was ≤20 ng/mL for 48% and ≤30 ng/mL for 89% of the entire year period, respectively.

Figure 1 (lower panel) graphically depicts the relative proportion of patients with serum 25(OH)D concentrations below the threshold values of 30 ng/mL, 20 ng/mL and 10 ng/mL as a function of the monthly period. February was the month with the highest prevalence of patients with serum 25(OH)D levels below 30 ng/mL (95%); April was the month with the highest prevalence of patients with serum 25(OH)D levels below 20 ng/mL (69%), and December the month with the highest prevalence of patients with serum 25(OH)D levels below 10 ng/mL (16%). 

Table 2 details the prevalence of serum 25(OH)D concentration below the three cutoff levels and according to the four trimesters. Considering the entire year, 6.5% of the population had serum 25(OH)D levels below 10 ng/mL, 40.1% below 20 ng/mL and 77.4% below 30 ng/mL. Relative proportions of patients with serum 25(OH)D concentration below all the three cutoff levels analyzed were significantly higher in the first year trimester compared to the third and fourth trimesters. On the other hand, data for the second trimester were similar to those reported for the first trimester for the 10 ng/mL and 20 ng/mL cutoff levels, while the rate of patients with serum 25(OH)D concentration below 30 ng/mL in the second trimester was significantly lower compared to first trimester.

**Figure 2:**
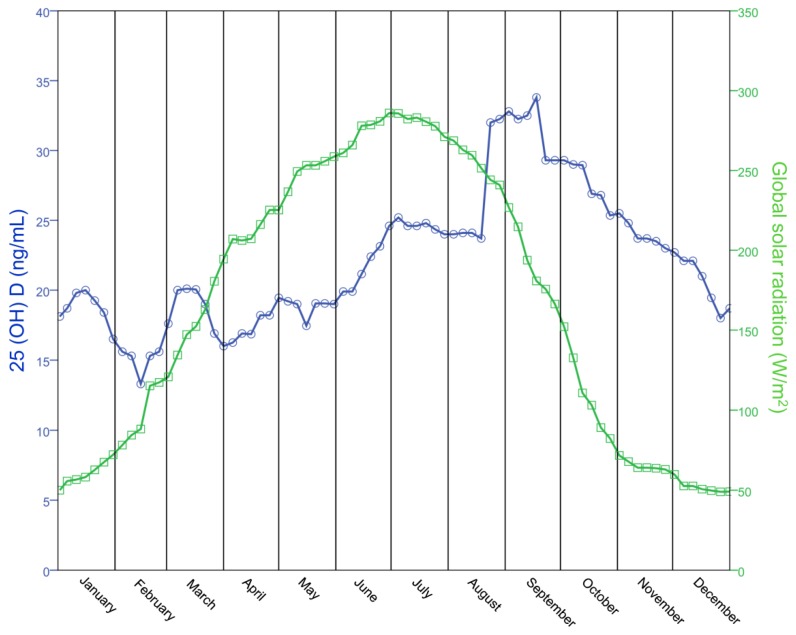
Relationship between solar radiation and 25(OH)D levels. Annual levels for global solar radiation is represented with green open squares and is compared to the moving median of 25(OH)D concentration (blue open circles).

Figure 2 graphically represents the moving median of global solar radiation in the Lombardy region during the year; as expected, a sine wave pattern was found where the maximum intensity was reached at the end of June (summer solstice) and the lower at the end of December (winter solstice). When global solar radiation during the year was compared to the moving median of 25(OH)D concentration values for patients living in the Lombardy region (*n* = 563), a weak correlation was observed. Throughout the first five months of the year, 25(OH)D levels appeared to be completely unaffected by the increased global solar radiation. Then, a rapid increase in 25(OH)D levels could be observed during the month of June with the median value that exceeded the 20 ng/mL value, reaching a plateau a few days after the summer solstice. This plateau could be observed until the first half of August, when the 25(OH)D median level reached for the first time a value higher than 30 ng/mL, a level maintained until the end of September, when the 25(OH)D median level started to decrease.

**Table 2:** Prevalence of serum 25(OH)D concentration below the threshold values of 10 ng/mL, 20 ng/mL and 30 ng/mL and according to the four trimesters. The first trimester was used as a reference group for odds ratio calculation. CI, confidence interval.

	*n*	Prevalence (%)	Odds Ratio	CI 95%	*p*-Value
**<10 ng/mL**					
Total	1072	6.5			
Trimester					
1	256	10.9	1.00		
2	292	8.6	0.76	0.43–1.35	0.38
3	249	0.8	0.07	0.02–0.28	<0.0001
4	275	5.5	0.47	0.24–0.90	0.03
**<20 ng/mL**					
Total	1072	40.1			
Trimester					
1	256	55.1	1.00		
2	292	54.5	0.98	0.70–1.37	0.93
3	249	18.9	0.19	0.13–0.28	<0.0001
4	275	30.2	0.35	0.25–0.50	<0.0001
**<30 ng/mL**					
Total	1072	77.4			
Trimester					
1	256	92.2	1.00		
2	292	84.6	0.47	0.27–0.81	0.008
3	249	58.6	0.12	0.07–0.20	<0.0001
4	275	73.1	0.23	0.14–0.39	<0.0001

### 3.2. Determinants of Vitamin D Status in Women Attending the Infertility Center

Selection of measured variables associated with vitamin D status by linear regression showed a significant negative association of serum 25(OH)D levels, as normalized according to dosage date (expressed as *Z*-score percentiles), with weight (*p* = 0.0001); conversely, a positive association was observed for height (*p* = 0.001) (Table 3). Women currently smoking more than 15 cigarettes/day were more likely to have low 25(OH)D levels, although this result was not statistically significant. Among the different causes of infertility, women with endometriosis were more likely to have high 25(OH)D levels (*p* = 0.01) as compared with those attending the center for male factor infertility, who have been used as control group as previously performed [24,25]. There was no relationship between 25(OH)D levels and age (*p* = 0.90), antral follicles count, type of infertility, and hormonal levels. Multivariable linear regression models confirm the association of serum 25(OH)D levels with weight (*p* = 0.0003) and height (*p* < 0.0001). Moreover, endometriosis remained positively associated with 25(OH)D levels (*p* = 0.03). To better investigate the relationship among weight, height and 25(OH)D concentration, a multivariable stepwise regression was conducted using BMI, weight and height as independents variables for predicting vitamin D status. The results showed that BMI (B = −1.29, 95% CI from −1.77 to −0.81, *p* < 0.0001) and height (B = 0.38, 95% CI from 0.11 to 0.66, *p* = 0.007) were independently correlated with 25(OH)D levels, while weight was excluded from the model.

**Table 3:** Linear regression analysis results for measured variables as determinants of vitamin D status.

	Univariable				Multivariable			
	B	B 95% CI		*p*-Value	B	B 95% CI		*p*-Value
		Lower Bound	Upper Bound			Lower Bound	Upper Bound	
Age (Years)	−0.02	−0.42	0.37	0.90	−0.10	−0.67	0.48	0.74
**Weight (Kg)**	**−0.33**	**−0.50**	**−0.16**	**0.0001**	**−0.43**	**−0.66**	**−0.20**	**0.0003**
**Height (cm)**	**0.46**	**0.18**	**0.74**	**0.001**	**0.92**	**0.57**	**1.27**	**<0.0001**
Current smoking (No. cigarettes/day)								
0	0							
1–15	2.89	−1.85	7.62	0.23				
>15	−7.08	−14.49	0.34	0.06				
FSH (IU/L)	0.36	−0.06	0.79	0.09				
LH (IU/L)	0.39	−0.20	0.98	0.19				
E2 (pg/mL)	−0.11	−0.26	0.04	0.16				
PRL (ng/mL)	−0.11	−0.52	0.30	0.60				
TSH (µU/mL)	−0.11	−2.53	2.32	0.93				
AMH (ng/mL)	−0.02	−0.88	0.83	0.96				
Antral follicles count (*n*)	0.23	−0.21	0.67	0.31				
Type of infertility								
Primary	0							
Secondary	−2.01	−5.82	1.80	0.30				
Cause of Infertility								
Male factors	0				0			
Tubaric factors	−4.94	−13.43	3.54	0.25	−6.23	−14.75	2.28	0.15
Poor ovarian response	3.24	−3.44	9.93	0.34	1.50	−5.24	8.25	0.66
Polycystic OvarianSyndrome	4.49	−1.95	10.93	0.17	3.87	−3.37	11.10	0.29
**Endometriosis**	**10.21**	**2.06**	**18.36**	**0.01**	**8.93**	**0.73**	**17.14**	**0.03**
Idiopathic	5.18	-0.95	11.31	0.10	3.24	-2.98	9.45	0.31

Bold font represents statistically significant results. Coefficients (B) resulting from the regression analysis are expressed as the difference in normalized vitamin D concentration (*Z*-scores) percentiles due to a change of one unit in the independent variable. Coefficients for categorical variable are compared to the reference category indicated by B equal to 0. FSH, follicle-stimulating hormone; LH, luteinizing hormone; E2, estradiol; PRL, prolactin; TSH, thyroid-stimulating hormone; AMH, anti-müllerian hormone; CI, confidence interval.

## 4. Discussion

The results of this study indicate that in a cohort of female patients attending an infertility center in Northern Italy, levels of serum 25(OH)D follow a seasonal cycle; as a whole, over the entire year, 40.1% of patients showed deficient, and 77.4% insufficient or deficient levels, of 25(OH)D. The median value of serum 25(OH)D concentration reached a sufficient level only for 11% of the entire year period. This means that, excluding a period of 40 days between August and September, during the entire year the majority of women referring to an ART procedure showed insufficient vitamin D status. These findings are of clinical significance considering the recent evidence supporting a critical role of vitamin D in regulating human fertility [26]. Indeed, *in vitro* studies tend to suggest that there might be a relationship between vitamin D deficiency and granulosa cell function [27]. Vitamin D treatment has been shown to act in these cells down-regulating FSH receptor and antimullerian hormone (AMH) receptor II gene expression and increasing 3β-hydroxysteroid dehydrogenase expression and progesterone production. The favorable effects of vitamin D on the metabolic alterations in PCOS represented by decrease in insulin resistance and androgen levels have been suggested to be translated into a healthier ovarian physiology [2,27]. The relationship between 25(OH)D levels and *in-vitro* fertilization (IVF) outcomes has been inconsistent in the literature mostly for the multitude of factors involved such as sperm quality and endometrial receptivity and for the retrospective nature of the studies [2]. Our prospective cross-sectional investigation has demonstrated that vitamin D deficiency negatively affects ART clinical pregnancy rate, potentially opening new therapeutic scenarios for women scheduled for IVF and, further, in general, to all women with infertility [1]. However, evidence for a causative effect of 25(OH)D levels on IVF outcomes are still poor and studies conducted so far rarely meet all the Hill’s criteria for causation [28]. An increase in the number of more specific and consistent studies and the demonstration of the existence of a biological gradient of effect for different 25(OH)D levels will be needed in order to confirm previous observations.

Current findings may have major implications both for infertile patients and in terms of national healthcare policy. Since a large proportion of women attending an infertility center show insufficient circulating levels of 25(OH)D, physicians in Reproductive Medicine should consider to monitor serum 25(OH)D levels during the course of any ART cycle. In any case, no evidence of significant decrease in 25(OH)D concentration was found among the various causes of infertility when compared to the male factor group, which was considered a referral cohort that represented the general population since it included healthy women only. Evidence from randomized controlled trials is required to definitively support the benefit of vitamin D supplementation as a simple and inexpensive intervention to positively impact on pregnancy outcomes.

At the national healthcare level it has to be considered that there is a notable discrepancy between mean intakes and dietary recommendations among European countries. In this context, while in Scandinavian countries the consumption of vitamin D with the diet is high, both fortification and supplementation policies have also been implemented. In Germany, the emphasis is on encouraging outdoor sun exposure. Turkey has an infant supplementation program. Conversely, both Spain and Italy do not have formal fortification or supplementation public health policies. This results in lowest mean daily intakes in Spain and Italy compared to Scandinavian countries [29]. Prevalence of vitamin D deficiency among European population subgroups is difficult to be estimated but it should be considered that in a study population of 58 Italian healthy women (mean age equal to 36.9 years) the mean 25(OH)D serum levels were 15.2 and 30.7 ng/mL in winter and summer, respectively, with prevalence of 25(OH)D concentration lower than 20 ng/mL ranging from 81% in winter to 6.9% in summer [30,31]. Whether vitamin D status will prove to be important for fertility outcomes, the adoption of policies aimed at preventing vitamin D deficiency should be considered as a public health priority, and childbearing couples as a vulnerable group in the population.

The effect of seasonality on 25(OH)D levels is highlighted in this study. Median level of serum 25(OH)D was lower in the first trimester of the year, with a consequent increase in the prevalence of deficiency. These findings support previous results and highlight that season is a major factor in determining vitamin D status [32,33]. Moreover, we showed that global solar radiation was weakly correlated with 25(OH)D levels. In this context, it should be considered that Italian residents spent more than half of their tourism nights in July and Augusts, as recently reported by Eurostat [34]. This is supported by the vitamin D peak observed during August. Thus, the weak correlation observed with solar global radiation could be explained by the interference of social factors.

The association between 25(OH)D levels and age has produced controversial results, with some studies supporting an increased prevalence of deficiency with age and others failing to confirm this finding [35]. Our findings did not support an association between age and 25(OH)D concentration. However, this may be explained by the restricted age range of our cohort of women. 

Body weight was negatively associated with vitamin D status and this result is generally consistent with those identified in previous studies [36]. Our analysis has also evidenced a significant positive correlation between height and 25(OH)D level, which was confirmed at multivariable analyses. This intriguing effect has already been reported for young women in two studies [37,38] but still remain unexplained. In fact, vitamin D is key to skeletal development in childhood and its deficiency may result in short stature associated with rickets [39] but it is difficult to explain how this association could persist in adulthood. A possible explanation is that causes for vitamin D deficiency in childhood might perpetuate over the period of adulthood, but further investigation conducted in adult population are certainly needed.

Interestingly, among the causes of infertility, patients with endometriosis were found to have higher levels of 25(OH)D compared to women with other disorders. These findings are in line with the results of a prospective cohort study from our group demonstrating higher levels of 25(OH)D among reproductive-aged women selected for surgery for gynecologic indications [40]. On the other hand, they are in contrast with data from Harris *et al.* [41] who calculated a score for predicted 25(OH)D levels in 1385 cases of laparoscopically-confirmed incident endometriosis. Predicted plasma 25(OH)D level was inversely associated with endometriosis. Women in the highest quintile of predicted vitamin D level had a 24% lower risk of endometriosis than women in the lowest quintile [41]. Thus, the relationship between endometriosis and the vitamin D endocrine system still remains to be clarified. Although vitamin D deficiency may be revealed as a biologically plausible pathway to an increased risk of autoimmune and inflammatory diseases, such as endometriosis, because vitamin D is able to affect several arms of the immune function, genetic and/or epidemiological factors cannot be excluded in the interpretation of these data. Notably, the common phenotype of women with endometriosis might represent a factor predisposing to a higher vitamin D synthesis from the skin [42]. In fact, it has been demonstrated that the length of time required for endogenous vitamin D3 biosynthesis increases with increasing melanin concentration in the skin [43,44]. Women with endometriosis frequently show a typical fair skin phenotype [42]. Despite different interpretations in this context [45], this phenotype might probably be the cause of an increased vitamin D synthesis, thus resulting in higher 25(OH)D levels when compared with women with darker skin phenotype from the Italian general population.

Our study is not devoid of limitations. First, the cross-sectional design may limit casual inference on the effects of seasons and infertility causes on vitamin D status. Second, we did not administer a food-recall questionnaire or a questionnaire on vacation habits. However, as already mentioned, the majority of the Italian population spends their vacation during the months of July and August. Third, our results were derived from a single center in Northern Italy; therefore we are uncertain that our results are generalizable to other infertility centers across the country. Finally, the infertility center was located around latitude 45°28′ N thus, it is not certain whether similar results would be obtained in centers in other geographical locations.

One of the strengths of our study is that it is the first to address the prevalence of vitamin D deficiency in a female population attending an infertility center. We had a fairly large cohort to enable us to detect differences between the groups of interest. Indeed, our cohort contained a fair representation of the main indications to infertility treatment, thus enabling us to investigate the effects of various causes on vitamin D status.

Lastly, vitamin D insufficiency has been recently associated with an increased risk of gestational diabetes, pre-eclampsia, and small for gestational age infants [11]. The high prevalence of vitamin D deficiency in infertile women referring to an ART procedure is likely to be mirrored by a similar prevalence during pregnancy. Furthermore, in general, the demonstration of an advantage of adequate levels of vitamin D in IVF cycles would be strengthened by the potential subsequent advantage also in terms of prevention of obstetrics complications. Even if evidence-based data supporting the benefits of vitamin D supplementation before pregnancy are not yet available, this aspect needs to be carefully considered when approaching our data. 

## 5. Conclusions

We observed a high rate of hypovitaminosis D among childbearing women. Current findings show that 25(OH)D circulating levels seasonally fluctuate in a homogenous cohort of women seeking medical help for couple’s infertility in the northern part of Italy. Likewise, vitamin D status is associated with specific causes of infertility and individual anthropometric characteristics. The use of supplementation remains an issue in this population and reproductive physicians should consider this aspect in their clinical practice.
